# Weather variation affects the dispersal of grasshoppers beyond their elevational ranges

**DOI:** 10.1002/ece3.7045

**Published:** 2020-12-02

**Authors:** Andrew J. Prinster, Julian Resasco, Cesar R. Nufio

**Affiliations:** ^1^ Yale University New Haven CT USA; ^2^ Department of Ecology and Evolutionary Biology University of Colorado Boulder CO USA; ^3^ University of Colorado Museum of Natural History University of Colorado Boulder CO USA; ^4^ Howard Hughes Medical Institute Chevy Chase MD USA

**Keywords:** climate change, dispersal, elevational gradients, range expansion, range limits, source–sink dynamics

## Abstract

1. Understanding how abiotic conditions influence dispersal patterns of organisms is important for understanding the degree to which species can track and persist in the face of changing climate.
2. The goal of this study was to understand how weather conditions influence the dispersal pattern of multiple nonmigratory grasshopper species from lower elevation grassland habitats in which they complete their life‐cycles to higher elevations that extend beyond their range limits.
3. Using over a decade of weekly spring to late‐summer field survey data along an elevational gradient, we explored how abundance and richness of dispersing grasshoppers were influenced by temperature, precipitation, and wind speed and direction. We also examined how changes in population sizes at lower elevations might influence these patterns.
4. We observed that the abundance of dispersing grasshoppers along the gradient declined 4‐fold from the foothills to the subalpine and increased with warmer conditions and when wind flow patterns were mild or in the downslope direction. Thirty‐eight unique grasshopper species from lowland sites were detected as dispersers across the survey years, and warmer years and weak upslope wind conditions also increased the richness of these grasshoppers. The pattern of grasshoppers along the gradient was not sex biased. The positive effect of temperature on dispersal rates was likely explained by an increase in dispersal propensity rather than by an increase in the density of grasshoppers at low elevation sites.
5. The results of this study support the hypothesis that the dispersal patterns of organisms are influenced by changing climatic conditions themselves and as such, that this context‐dependent dispersal response should be considered when modeling and forecasting the ability of species to respond to climate change.

## Graphical abstract

Understanding how abiotic conditions influence dispersal patterns of organisms is important for understanding how species track and persist in the face of changing climate. The goal of this study was to understand how weather conditions influence the dispersal pattern of multiple grasshopper species from lower to higher elevations that extend beyond their range limits. The results of this study support the hypothesis that the dispersal patterns of organisms are influenced by changing climatic conditions themselves and as such, that this context‐dependent dispersal response should be considered when modeling and forecasting the ability of species to respond to climate change.

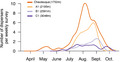

The continual wide dissemination of so‐called accidentals [dispersers], has, then, provided the mechanism by which each species as a whole spreads, or by which it travels from place to place when this is necessitated by shifting barriers. They constitute sort of sensitive tentacles by which the species keeps aware of the possibilities of a real expansion. In a world of changing conditions, it is necessary that close touch be maintained between a species and its geographical limits, else it will be cut off directly from persistence… Grinnell (1922)

## 1 INTRODUCTION

Dispersal is an important process that influences the spatial and temporal distributions, local stability, and genetic structure of populations (Clobert et al., 2012; Ibrahim et al., 1996). As dispersal affects the evolutionary dynamics of spatially structured populations, it is an important life‐history trait that impacts the ability of populations to respond to environmental change (Bell & Gonzalez, 2011; Bonte & Dahirel, 2017; Ronce, 2007). The dispersal pattern of organisms is relevant not only in understanding how populations respond and adapt to changing local conditions, but also in the context of whether species will be able to spatially track shifting conditions that reflect their thermal preferences under climate change (Bonebrake et al., 2018; Halbritter et al., 2013; Loarie et al., 2009; Maguire et al., 2015; Malcolm et al., 2002; VanDerWal et al., 2013). That is, dispersal patterns of species (influenced by their dispersal propensity, flight ability, and thermal limitation) may influence whether and to what degree they are able to persist in the face of changing local and regional climates by shifting their distributions into more favorable areas (Bonebrake et al., 2018; Buckley et al., 2013; Driscoll et al., 2014; Urban et al., 2016).

Abiotic factors are known to alter dispersal patterns directly through their impacts on species (influencing their developmental trajectories, physiologies, and dispersal propensity) or indirectly through their influences on the biotic and abiotic environment (availability of microhabitats and hosts, and influencing species interactions) (Le Galliard et al., 2012). Changes in temperature, precipitation, and other weather factors (e.g., wind speed), are known to influence dispersal patterns, although the strength and direction of effect can be highly taxon dependent (Travis et al., 2013). In the context of climate change, if species’ dispersal patterns themselves are impacted by weather variability and changing climatic conditions (Bowler & Benton, 2005; Doerr et al., 2011; Hodgson et al., 2009; Travis et al., 2013), this challenges the assumption that dispersal potential is a fixed trait (Clobert, 2001; Record et al., 2018; Ronce, 2007; Thomas et al., 2003; Thuiller et al., 2008). Given the likelihood that dispersal is context‐dependent rather than a fixed trait, climate change studies should invoke a more dynamic expectation of how species' dispersal responses will themselves be influenced by shifting climatic conditions (Kokko & Lopez‐Sepulcre, 2006; Travis et al., 2013).

In this study, we investigate how shifting climatic conditions may influence the rate at which species disperse beyond their elevational range limits. More specifically, we examine how variation in temperature, precipitation, and wind speed and direction influence the dispersal patterns of an assemblage of low elevation nonmigratory grasshoppers along an elevational gradient in the southern Rocky Mountains (Colorado, USA). To quantify changes in dispersal patterns along this gradient, we conducted weekly spring to late‐summer surveys at foothill to subalpine sites and recorded the abundance and species richness of grasshoppers that dispersed to these sites from the high plains. While montane grasshoppers are considered residents when they complete their life‐cycles at higher elevations, likely due to an ability to meet their physiological requirements, adult grasshoppers dispersing from lowland a montane areas are considered non‐residents because they are restricted to initiating and completing their full life‐cycles at lower elevations (Alexander, 1964). Here, we refer to these non‐resident individuals and species as “dispersers,” while other terms used may include “vagrants” and “accidentals.” These disperser species may be relatively rare or common at montane sites depending on year and site and the net movement of these grasshoppers is in the upslope direction (Alexander & Hilliard, 1969). In this study, we make no assumptions about whether dispersing grasshoppers are passively displaced or actively disperse to sites in response to shifting weather patterns.

Grasshoppers are appropriate organisms for exploring how abiotic conditions influence dispersal patterns because abiotic conditions impact their development, daily activity patterns, movement behaviors, and population dynamics (Bale et al., 2002; Beck, 1983; Buckley et al., 2014; Chappell & Whitman, 1990; Jonas et al., 2015; Olfert & Weiss, 2006). Changes to grasshopper dispersal patterns are also of interest broadly because the dominance of these herbivores in grassland ecosystems means that large scale changes in their movement patterns can have important impacts on ecosystem, rangeland, and agricultural systems (Branson et al., 2006).

In this study, we test the hypothesis that the dispersal propensity of organisms beyond their range limits can be context‐dependent and, in particular, that it is influenced by changing weather conditions. Given previous work on grasshoppers, if dispersal responses vary with abiotic conditions, we predict that warmer temperatures and lower precipitation levels should increase the abundance and richness of dispersing grasshoppers from lower to higher elevations (Alexander, 1951, 1964; Walters et al., 2006). However, based on the literature, it is unclear whether we should expect that the directionality and strength of east‐west wind patterns, that move from lower to higher elevation along the east sloping mountain, should increase (Alexander, 1951, 1964) or decrease (Narisu et al., 2000) the rate at which individuals actively or passively disperse along the gradient. We also explore whether the dispersal propensity of these grasshoppers differs between males and females. Finally, by examining the population dynamics (abundance) of resident grasshoppers at the lowest elevation site and its relationship with weather conditions, we propose and address the relative importance of two mechanistic hypotheses that could explain the flow of dispersing individuals. The first hypothesis proposes that an increase in the number of dispersers found at higher elevations is due to a net increase in the number of grasshoppers at lowland source sites that may be promoted by warmer and drier conditions. The second hypothesis proposes that weather conditions directly or indirectly influence the dispersal propensity of grasshoppers rather than increase the number of potentially available dispersers. A correlation between weather conditions that lead to increases in grasshopper abundance and the number of sampled dispersers would suggest that changes in abiotic conditions promote dispersal through their positive impact on lowland population dynamics. However, if the weather conditions that promote dispersal patterns are opposed to or do not reflect the conditions that promote grasshopper population sizes, increased movement rates of grasshoppers would be best explained by changes in dispersal propensity that is influenced by changing abiotic conditions.

## 2 MATERIALS AND METHODS

### 2.1 Field sites and grasshopper surveys

**FIGURE 1:**
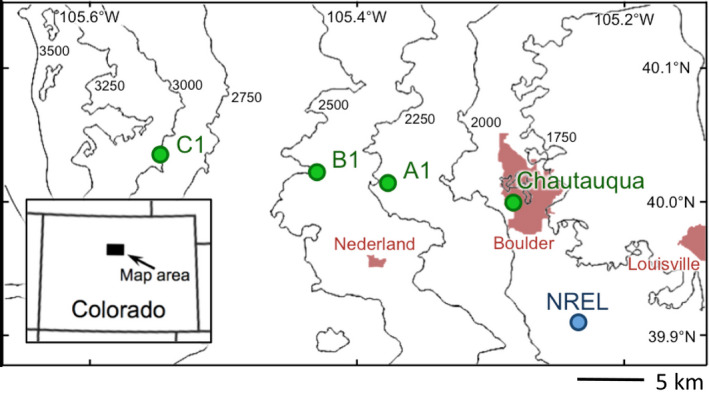
Map denoting surveyed sites (Chautauqua mesa, A1‐C1) along the elevational gradient in Boulder County, Colorado as green circles. The NREL weather station is indicated by a blue circle. Topographic curvature lines delineate changes in elevation (m). Cities and towns and included for reference in pink

Grasshoppers in this study were sampled from four sites along an approximately 1,300‐m elevational gradient within Boulder County, Colorado, USA. These sites, running along the 40th parallel, are Chautauqua Mesa (1,752 m), A1 (2,195 m), B1 (meadow west of) (2,591 m), and C1 (3,048 m) and reflect southeast‐facing grassy clearings associated with distinct life‐zones (Table 1, Figure 1). As one moves from the foothills to the sub‐alpine life zones, there is an increase in the average total precipitation (52.5–67.0 cm) and a decrease in the average yearly temperature (10.5–1.7°C) (Kittel et al., 2015; McGuire et al., 2012; Western Regional Climate Center, 2020).

**TABLE 1:** Location and description of surveyed sites along the elevational gradient

Site	Elevation (m)	Latitude	Longitude	Life zone classification	Number of years surveyed^a^	Number of collecting events^b^
Chautauqua Mesa	1,752	39.999	−105.283	Foothills	8	111
A1	2,195	40.015	−105.377	Premontane	6	82
B1	2,591	40.023	−105.430	Montane	10	142
C1	3,048	40.036	−105.547	Sub‐alpine	11	140

^a^ Two of each of these years are historical surveys (except A1 which only has 1), and the rest are from contemporary surveys.

^b^ Total number of sampling events conducted at a site during the contemporary survey.

The grasshopper data used in this study were collected via weekly surveys conducted during two time periods constituting historic, 1958–60 (Alexander & Hilliard, 1969) and contemporary surveys, 2006–15 (Nufio & Buckley, 2019). In particular, the historic and contemporary surveys were combined to document the occurrence of dispersing species and their abundance, and to examine the role of precipitation and temperature on grasshopper population sizes at Chautauqua Mesa. However, only the contemporary survey data were used to explore the effects of weather variables on dispersal patterns because corresponding wind data do not exist for the historical survey years. Because of a lack of consistency in the frequency of weekly surveys in 1958 and a lack of effort to document dispersers in 2006 (the first year of each survey period), these years were not included in the analyses, providing a 6‐ to 11‐year data survey record depending on the site (Table 1).

As the field season for adult grasshoppers begins in spring and ends in late summer, the weekly historic and contemporary surveys began in May or June (depending on the initiation of the season due to elevation) and extended into mid‐September. While the continuous open grassy areas associated with the surveyed sites vary in size and degrees of isolation, the surveys for both time periods were standardized as follows (based on historic field notebook data and discussions with previous surveyors, D. Van Dorn and D. Alexander). Each survey consisted of systematic 1.5 person‐hours of sweep netting (divided among 1–3 surveyors) and 0.75 person‐hours of time spent searching for adults and juveniles (nymphs) that may have been missed by sweep netting. The area covered per survey varied depending on the abundance of grasshoppers and time of the season, but typically covered an area of 1.5–3 ha. During each survey, grasshoppers were processed in the field where their numbers, developmental stages, and sexes, and species designations were recorded. To minimize potential observer biases (due to differences in sweep‐netting techniques or ability to search and identify individuals), the same collector was involved and made the identifications during the current surveys (C. Nufio). In turn, a subset of grasshoppers that included common species and particularly unusual sightings were brought to the laboratory to document species and ensure proper identification. Thus, the likelihood that dispersing individuals were continuously resampled was considered low. Dispersers in this study are defined and restricted to the detection of adult grasshoppers of species that do not initiate and complete their life‐cycles at a given site. In other words, the absence of juveniles of these dispersing species at the sites suggests that these species have not become established. The majority of dispersing species detected along the gradient are only residents at lowland plains below the foothills (25 species making up 675 detected individuals), while the rest of the dispersing species (13 species making up 140 detected individuals) are not only resident at lowland sites but can also be found at similar or higher elevations as the sites they were detected in (Alexander, 1964; Alexander & Hilliard, 1969). Given that we do not tend to find species below their expected elevational gradients, we believed that the assumption that grasshopper dispersal is predominantly in the upslope direction is valid (Alexander & Hilliard, 1969). However, at Chautauqua Mesa, the lowest elevation site, two species previously considered residents by Alexander (1964) (*Ageneotettix deorum* and *Melanoplus packardii*) are currently considered non‐resident dispersers to the site because five contemporary survey years have only detected a few adults of these species and have not detected their juveniles even though over 18 thousand grasshoppers (including mostly juveniles) have been recently processed at this site (Nufio & Buckley, 2019). The reasons for the loss of these resident species (as well as 6 other species no longer detected as juveniles or adults at this site) are unknown but may reflect factors such as a decrease in habitat area (Nufio et al., 2009), the introduction of invasive grasses, and a reduction in the availability of open habitats. It is important to note that all eight non‐resident species at the site are commonly detected (as adults and juveniles) within Boulder county. Finally, at Chautauqua Mesa remaining resident species vary as to whether they are currently more or less common, and these patterns may reflect environmental changes, as well as, year‐to‐year weather variation (see *Population size and weather* section below).

Finally, in this study, we do not include as dispersers species referred to as transients by Alexander (1964); species that may complete their life‐cycles in montane sites on some years but not others (e.g., *Melanoplus sanguinipes* at B1 & C1 in both the historic and contemporary surveys, *M. bivittatus* found at C1 only in 1959).

### 2.2 Weather data

The temperature, precipitation, and wind data used for analyses corresponding to the 2007–2015 time period were obtained from the National Renewable Energy Laboratory's National Wind Technology Center's (NWTC) M2 Tower (1,855 m; 39.9106 N−105.2348 W; Jager & Andreas, 1996). The tower is located near the base of the Rocky Mountains near Boulder, Colorado and thus approximates the conditions experienced at lower elevations from which dispersing individuals originate (Figure 1). The raw weather and wind data used in the study and provided by the M2 tower includes measurements recorded every two seconds and averaged over one‐minute intervals for temperatures at a 2‐m height, and the wind speed and direction at 2 and 80‐m heights. The wind speed and directions at the two heights correspond to conditions experienced by grasshoppers when near the ground and when in flight (Chapman et al., 2004). Precipitation is provided by the M2 tower as the total accumulated since the beginning of a given day. For our analyses, calculations of the average daily temperature (°C), wind speeds (m/s), and wind components (see below) were restricted to those occurring from 6 a.m. to 6 p.m., the approximate hours of grasshopper activity. Calculations of accumulated precipitation (mm) were, however, based on daily totals. The average NREL temperature, precipitation, and wind vector data were calculated from June 1 to September 7 to largely match the time frame of the 11 collection events. In this study, only weather conditions at the base of the mountain were used to determine dispersal patterns because these conditions are expected to be most consequential for influencing the dispersal patterns of lowland species from their points of origin to higher elevations.

While wind speed serves as a metric of the overall magnitude of winds in any direction, wind vector components are used to account for both the speed and direction of winds. By convention, the speed and direction of any horizontal wind can be described by two orthogonal wind components, the zonal (west‐east) component *u* and the meridional (south‐north) component *v* (National Center for Atmospheric Research, 2013). These components are calculated as

$$\begin{array}{c} u=‐WS\cdot \sin\left(\frac{\pi \cdot \theta }{180}\right)\\ \text{and}\\ v=‐WS\cdot \cos\left(\frac{\pi \cdot \theta }{180}\right) \end{array}$$

where *WS* is the wind speed and *θ* is the direction the wind is coming from (degrees clockwise from north). When added together, these components produce a resultant vector whose magnitude represents the wind speed and whose direction points in the direction toward which the wind is moving.

Since upslope grasshopper dispersal is of interest to our study, we focus on wind in the east‐west direction which runs parallel to the direction of our elevational gradient. While the north‐south components may contribute to these movement patterns, given the complexity of wind data, the number of study sites and our inability to infer directionality of grasshopper flight patterns, we simply make the assumption that its orthogonal direction to the gradient is likely less relevant for upslope dispersal. Thus, the mean zonal (hereon U‐vector) component for any given time period can be interpreted as the mean wind flow from west to east over that time period (in m^3^/s through one m^2^ of area perpendicular to the direction of wind flow). Positive U‐vector values imply that the net movement of wind is from the west to the east (downslope), and negative U‐vector values indicate net wind movement is from the east to the west (upslope). The larger the values in the positive or negative direction, the greater the wind flow in that given direction. During morning and afternoon hours when grasshoppers are active, net daily U‐vector wind flow is typically in the upslope direction.

### 2.3 General dispersal patterns

To understand the relationship between the number of dispersers found at site and elevation, we summed the total number of individual dispersers and dispersing species detected at each of the four sites during the historical (1959 and 1960) and the contemporary (2007–2015) survey years (*n* = 35, Table 1). For this and subsequent analyses, we restricted each year's data on the number of non‐resident dispersers at each site to 11 collection dates that began in early June and ended on or just prior to September 7. Given that the initiation of field surveys at a given site reflected a natural delay in the start of the field seasons with elevation (beginning in May at the lowest elevation and June at the highest), this restriction ensured that each site was represented by an equal number of sampling events that were also conducted during the same time intervals each year. Although this strategy slightly underestimated the number of dispersers at the lower sites, these restricted collecting events well encompassed most grasshopper abundance and dispersal activity periods occurring over a season. Because sex‐biased dispersal can be important for range expansion (Miller & Inouye, 2013), we pooled and compared the number of male and female dispersers collected across sites using the combined historic and contemporary survey data.

### 2.4 Dispersal patterns and weather

To determine the effects of elevation, year, and weather variables on the number and species richness of dispersing grasshoppers collected during the contemporary surveys (2007–2015) we fit generalized linear models (GLM). In the models, we assumed a Poisson distribution and used a log link function. To determine which height (2 or 80 m) was most appropriate to use for wind variables, we used likelihood ratio tests (LRT) to compare the difference in deviance between a base GLM that included site elevation, temperature, precipitation, and year, with models that included the addition of each wind variable independently (wind speed or wind U‐vector at 2 or 80 m). We then fit models with the number of dispersing individuals as a response variable and the following as predictor variables: year, elevation, temperature, precipitation, a temperature x precipitation interaction, and wind speed and wind U‐vector variables at the heights with the greatest explanatory power. The predictor variables in all models were centered and scaled to allow for a comparison of the relative importance of their coefficients. We included year as a fixed effect to test for temporal trends in the data. We used variance inflation factors (VIFs) to assess collinearity among variables and removed problematic variables from the model. We did model selection using AICc to identify the most parsimonious model out of those made up of all combinations of predictor variables. The most parsimonious model was then used to examine the corresponding effects of elevation and weather variables on the number of species detected (richness) across the sites over the years. Diagnostic plots were used to evaluate the fit of the models and pseudo *R* ^2^’s for models were calculated (Zuur et al., 2009). In all models, elevation was treated as a continuous variable because such a designation provides quantitative information that can be used to build ecological models and because regression approaches have been shown to provide greater power for detecting changes along gradients and for quantifying responses to multiple factors (see Cottingham et al., 2005; Somerfield et al., 2002). We also subsequently fit generalized linear mixed models (GLMMs) with elevation and site as random effects and otherwise the same fixed effects as in the GLMs above to determine whether the results of weather variables were robust. To examine whether there was a departure from the expected 1:1 sex ratio in the dispersers collected along the gradient, we used chi‐square tests that pooled data across all sites and among the sites across all surveyed years.

### 2.5 Population size and weather

We used the lowest elevation foothills site, Chautauqua Mesa, as a proxy for understanding the relationship between the density of adult resident grasshoppers at lower elevations (from where the nonresident dispersers arise) and changes in the average temperature and cumulative precipitation that occurs from spring to summer. Chautauqua Mesa was used because it is nearest to the lowland sites and because we do not have long‐term survey data from the high plains area.

To estimate the yearly density of resident grasshoppers at Chautauqua Mesa during the 1959–1960 and 2007–2012 surveys, we tallied the total number of grasshoppers of 9 species that were residents during both time periods and were surveyed during the 11 collection dates that occurred from June 1 to September 7. These species were *Aeropedellus clavatus*, *Arphia conspersa*, *Eritettix simplex*, *Hesperotettix viridis*, *Melanoplus bivittatus*, *M. confusus*, *M. dawsoni*, *M. femurrubrum,* and *M. sanguinipes*. To calculate the average seasonal temperature and total precipitation associated with each surveyed year, we used the United States Department of Commerce's National Oceanic and Atmospheric Administration (NOAA) weather station data (Cooperative ID 050848; 39.9919–105.2667; data available via https://wrcc.dri.edu/) because the NREL station that collects current wind pattern data was established in the late 1970's and thus does not account for the temperature and precipitation levels associated with the 1959–1960 surveys. This NOAA weather station is located 1.3 km away from Chautauqua Mesa and is at a similar elevation (1671 m). Because May is the wettest month of the season and could have a large impact on the development of vegetation, we calculated the average temperature and total precipitation for each season from May 1 (a month earlier than the 11 survey periods were initiated) to September 7.

### 2.6 Population size and weather analysis

A multiple regression analysis was used to examine the relationship between the total number of resident grasshoppers collected at Chautauqua Mesa from June 1 to September 7, with the average seasonal temperature and total precipitation occurring from May 1 to September 7 from the NAAO's Boulder weather station. The predictor variables (temperature and precipitation) were centered and scaled to allow for a comparison of the relative importance of their coefficients. Interaction effects between precipitation and temperature to explain grasshopper numbers were explored and are presented. We used R version 3.4.1 (R Core Team, 2017), the *car* package (Fox & Weisberg, 2011) for calculating VIFs, and the MuMIn package (Bartoń, 2017) for model selection.

## 3 RESULTS

**FIGURE 2:**
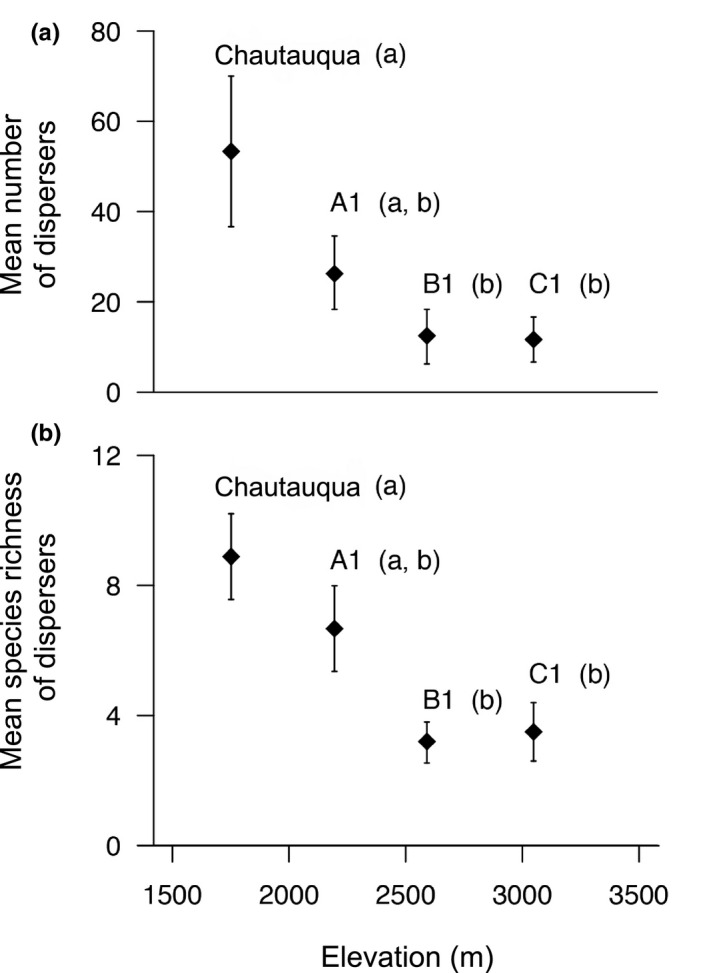
(a) The average number of dispersers per year by elevation. (b) The average species richness of dispersers found per site by elevation. Different letters associated with sites represent significant Tukey's HSD differences (*p* < .05)

**FIGURE 3:**
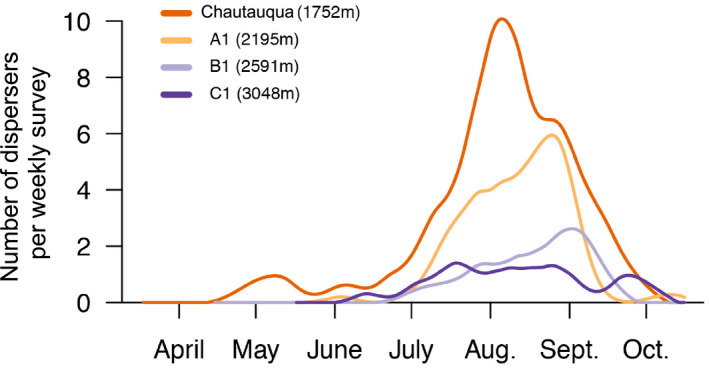
The number of dispersing grasshoppers detected during weekly surveys over a season at four sites along an elevational gradient. Lines are created from smoothing splines using the 2007–2015 data

When considering both the historical and contemporary surveys and restricting each surveyed year to 11 collection dates, 851 (684 from the contemporary survey) individual dispersers were detected along the elevational gradient, and these individuals represented 38 unique lowland prairie species. Along the mountain there was a decline in the average number of dispersers (Figure 2a; *F*_3,31_ = 3.94, *p* = .017, ANOVA) and their species richness per year (Figure 2b; *F*_3,32_ = 6.43, *p* = .0016, ANOVA) associated with increases in elevation. On a yearly basis, dispersers were four times more numerous and twice as species rich at the lowest site (Chautauqua Mesa) compared with the highest site (C1) which were associated with 52.13 (±*SE* 10) and 12.36 (±8.5) individual dispersers and 9 (±1.04) and 3.90 (±0.88) species per year, respectively. An analysis of the sex ratio of the grasshoppers collected across the sites did not show that dispersal was sex‐biased when the data were pooled across sites (*X* ^2^ = 0.08, *p* = .77) or when the pattern was examined at each site (Chautauqua Mesa, *X* ^2^ = 0.02, *p* = .88; A1, *X* ^2^ = 0.03, *p* = .85; B1, *X* ^2^ = 0.16, *p* = .69; C1, *X* ^2^ = 0.67, *p* = .41). Using the full survey data, the number of dispersers collected during a given survey date increased in mid‐summer (July) was greatest in early August and declined in September. The lower the elevation of the collection site, the greater the number of dispersers detected per survey and the earlier the maximum number of dispersers per survey was reached in the season (Figure 3).

### 3.1 Dispersal patterns and weather

**FIGURE 4:**
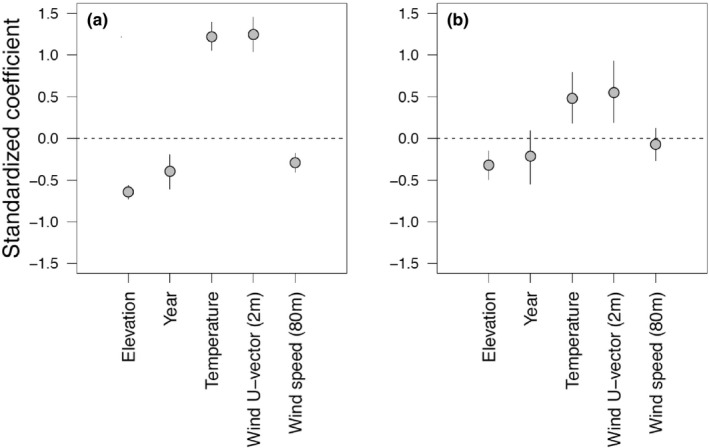
Standardized coefficients and 95% CIs from GLMs for *Elevation*, *Year*, and weather variables for (a) number of dispersers and (b) species richness of dispersers response variables

Over the surveyed years, wind speed at 80‐m and wind U‐vector at 2‐m height resulted in the greatest change in deviance compared with other heights. Our initial full model thus had disperser individuals as a response variable and as predictor variables: year, elevation, temperature, precipitation, a temperature x precipitation interaction, wind speed (80‐m), and wind U‐vector (2‐m). VIFs for the full model indicated that the inclusion of the temperature x precipitation interaction and precipitation variables (VIF = 10.0 and 11.9, respectively) resulted in co‐linearity in the model. Dropping these terms resulted in VIF values of 5.3 or lower. Zuur et al. (2009) state that such values, well below 10, suggest collinearity is not a major issue. We note that temperature and precipitation were inversely related (*R* ^2^ = .27, *p* = .004), such that warmer years tended to be drier and vice‐versa and that this correlation makes it difficult tease apart the independent effects of temperature and precipitation on grasshopper dispersal patterns. Model selection indicated that the full model with all remaining predictor variables (year, elevation, temperature, wind U‐vector, wind speed) was the most parsimonious compared with all other candidate models (AICc = 283.4, 13.15 AICc values lower than the next model, weight = 0.999). The number of dispersers declined with elevation and increased with temperature (Table 2a). The net daily seasonal wind flow of grasshoppers tended to be in the upslope direction and the number of dispersers increased with increasing U‐vector values, indicating more dispersing individuals are found at sites during calmer or downslope wind conditions (Table 2a). The number of these non‐residents also declined as wind speeds (80‐m) at the base of the mountain increased and a negative effect of year was detected (Table 2a, Figure 4a). This full model accounted for 84% of the variance (pseudo *R* ^2^) in the number of dispersers detected over the season. Effects of weather variables on species richness of these individuals were detected for only temperature and wind U‐vector (Table 2b). The coefficients showed the same direction of effects as those for the number of dispersers in the model (Table 2, Figure 4b) as the number of dispersing individuals collected at a site each year was correlated with species richness (*R* ^2^ = .64, *p* « .0001). This model accounted for 50% of the variance (pseudo *R* ^2^). Modeling elevation (site) and year as random effects in GLMMs resulted in generally similar results on the effects of weather variables as GLMs (Table 1, Table S1).

**TABLE 2:** Full‐season model summaries examining relationship between weather and other variables on (a) the number and (b) species richness of dispersing grasshoppers detected over a full season

	Standardized coefficient	*SE*	*z*‐value	*p*‐value
(a)				
Elevation	−0.64	0.04	−15.32	<2 × 10^–16^
Temperature	1.22	0.09	14.14	<2 × 10^–16^
Year	−0.40	0.10	−3.78	2 × 10^–4^
Wind U‐vector (2 m)	1.22	0.11	11.75	<2 × 10^–16^
Wind speed (80 m)	−0.29	0.06	−5.05	4.46 × 10^–7^
(b)				
Elevation	−0.32	0.09	−3.65	.0003
Temperature	0.48	0.15	3.10	.002
Year	−0.21	0.16	−1.32	.190
Wind U‐vector (2 m)	0.55	0.19	2.93	.003
Wind speed (80 m)	−0.07	0.10	−0.73	.470

### 3.2 Population size and weather

At Chautauqua Mesa, the total number of grasshoppers collected each year over the 1959–1960 and 2007–2012 surveys was negatively related to increases in temperature (*F*_1,1_ = 20.77, Standardized Coefficient (SC) = −195.44, *p* = .01). While precipitation itself did not explain changes in resident grasshopper densities (*F*_1,1_ = 3.07, SC = 9.25, *p* = .15), an interaction between temperature and precipitation (*F*_1,1_ = 16.38, SC = −427.93, *p* = .015) showed that reductions in the number of residents due to increases in temperature were stronger on drier years (model summary: *F*_3,4_ = 13.41, *p* = .01, *R* ^2^ = 0.91).

## 4 DISCUSSION

Quantifying the dispersal potential of organisms and how weather conditions influence these patterns is essential for understanding how changing climates themselves may positively or negatively influence the ability of organisms to modify their distributions and persist (Bonebrake et al., 2018; Hickling et al., 2006; Travis et al., 2013). In this study, we quantified the collective dispersal pattern of 38 unique grasshopper species that originated primarily from highland prairies and moved along a foothills to subalpine gradient. As expected, the number of dispersing individuals and species found at a site decreased with the site's distance from their low elevation source populations (Nathan et al., 2012). In particular, from the foothills to the subalpine there was a fourfold decrease in the number of dispersers and a 2.5‐fold decrease in the number of dispersing species detected over a season. Although some studies have shown that sex‐biased dispersal can be important in grasshoppers (Walters et al., 2006) and other insects (Albrectsen & Nachman, 2001; Miller & Inouye, 2013; Mishra et al., 2018), we did not detect a biased sex ratio among the dispersing grasshoppers that were collected.

Across years, the most parsimonious and best supported model showed that the number of dispersers and dispersing species detected at sites was, as predicted, positively related to increases in seasonal temperatures. However, due to the strong co‐linearity between precipitation and temperature and the removal of precipitation from the best supported full model, it was not possible to explore the additional or potentially consequential effects of precipitation. Larger data sets, longer times series, and future experimental approaches may help untangle the potential independent role of precipitation on these dispersal patterns. Other studies have found that warmer and drier weather patterns can both lead to increases in the dispersal of insects beyond their normal ranges (Parmesan, 2006). Still, the measured increase in the dispersal rates of grasshoppers with yearly increases in temperature is consistent with studies that suggest a long‐term warming pattern is the main driver of recent range expansions of grasshoppers and other orthoptera across temperate Europe (Fumy et al., 2020; Poniatowski et al., 2018). Unlike studies suggesting that grasshoppers may disperse against (Narisu et al., 2000) or with (Alexander, 1951, 1964) the dominant wind flow directions, our study found that decreases in the velocity of airflow at low elevation in the upslope direction led to increases in the movement of grasshoppers along the gradient. These findings are consistent with a large‐scale study which found that high wind current velocities reduced the flow of insects on a regional scale, while moderate wind flow rates and warmer days promoted the dispersal of larger diurnal insects (Hu et al., 2016). As the greatest number of dispersers were collected during several years when the predominant wind flow patterns where mild and in the downhill direction, this suggests that the effect of wind velocity and direction on grasshopper dispersal patterns may be more nuanced than whether insects simply move toward or away from dominant wind patterns. Although it is not clear how wind flow patterns may change with future global warming scenarios (Pryor & Barthelmie, 2011), their role in influencing species interactions and in supporting the movement of a variety insects, plants and other organisms is important (Barton, 2014; Pasek, 1988; Stinner et al., 1983) and should be considered, along with other weather associated patterns, when modeling future dispersal patterns (see, for example, La Sorte et al., 2019).

Examination of the flow dispersers along the gradient over each season is not uniform, rather it is composed of distinct temporal pulses. That is, the peaks in the number of dispersing grasshoppers are staggered, with the highest number being detected at the lowest site (Chautauqua Mesa) in mid to late‐July and the peak occurring in early September in the upper montane (B1) (Figure 3). As *Amphitornus coloradus* and *Trachyrhachys kiowa* are among the two most commonly collected dispersing species at each of the sites, the temporal staggering in peak abundances could reflect a progressive wave of lowland dispersers moving higher and higher along the mountain.

At the lowest site, Chautauqua Mesa, the abundance of resident grasshoppers collected over a season was explained by an interaction between the average seasonal temperature and precipitation. That is, the strong negative relationship between warmer seasonal temperatures and grasshopper abundance was steeper when precipitation levels were lower. Numerous long‐term studies on grasshopper population dynamics in grassland ecosystems suggest that a variety of factors, such as the previous season's grasshopper densities, and previous and current seasonal temperature and precipitation patterns can explain grasshopper population dynamics (Branson et al., 2006; Fielding & Brusven, 1990; Jonas et al., 2015; Yu et al., 2009). The relative strength and directions of the relationships between grasshopper densities and weather conditions in these studies appear to differ between populations found at different latitudes and sites (where the base temperatures and precipitation levels may differ) and between species. If population dynamics and their correlations with weather patterns at Chautauqua Mesa are similar to those at other lowland sites, the detection of a greater number dispersing individuals along the mountain on warmer (and on potentially drier) years may be best explained by changes in the dispersal rates of grasshoppers rather than to an increase the number of potential dispersers (Amarasekare, 2004; Matthysen, 2005).

While this study is based on extensive weekly surveys conducted for over a decade at four sites, and these sites have been used previously to detect phenological (Nufio & Buckley, 2019; Nufio et al., 2010) and phenotypic cline (Buckley et al., 2013; Levy & Nufio, 2015) patterns along an elevational gradient, the ability to infer large scale dispersal patterns is limited because each elevation is represented by a single site. Given this caution, if this study reflects larger scale patterns, the results suggest that warmer years and mild wind conditions can promote the propensity of a large assemblage of lowland grasshopper species to disperse along elevational gradients. If regional climates continue to warm (Maguire et al., 2015), become drier (Cook et al., 2015), and wind speeds decline (Karnauskas et al., 2018), and this leads to a consistent increase in the dispersal of grasshoppers in an upslope direction, this could have a variety of implications. First, an increase in dispersal propensity may allow these herbivores, and perhaps other insects, to more effectively track the rate of changing environmental conditions (Fumy et al., 2020; Poniatowski et al., 2018). Although these current dispersers are not able to persist at the sites they immigrate to, they represent potential colonizers should future conditions become less conducive to their growth and survival at lower elevations and should higher elevations become more hospitable (Grinnell, 1922). The number of dispersers detected through brief weekly and hourly surveys and within relatively restricted survey areas suggests that this movement of lowland grasshoppers and other insects across a mountain system could be significant and even influence ecosystem level processes (see Hu et al., 2016 for large scale estimates). Changes in the dispersal pattern of organisms associated with changing weather conditions also have implications for the range expansions of invasive species (Morrison et al., 2005). Second, although this current study focused primarily on dispersing species that are not residents at higher elevations, if changing environmental conditions promote the dispersal of species with more extensive low to high elevation ranges, these increased dispersal rates may increase gene flow patterns which could have implications for populations, their degrees of local adaptation and their ability to respond to future warming (Clobert et al., 2012; Larson et al., 2019; Levy & Nufio, 2015). Finally, while not explored in this study, a future examination of the dispersing species collected in this study, as well as their among species variation, could inform our understanding of how species traits (such as body sizes and morphology, and degrees of phenotypic plasticity), seasonal timing patterns, thermal sensitivities, and interactions with other species may influence the differential dispersal rates of species moving along elevational and latitudinal gradients (Bonebrake et al., 2018; Buckley et al., 2013; Buse & Griebeler, 2011; Cale, 2003; Clobert et al., 2012; De Bie et al., 2012; Matthysen, 2005; Padial et al., 2014; Van der Putten et al., 2010; Wang et al., 2018; Zera & Denno, 1997). Further monitoring and a closer look at the traits of species most likely to disperse would inform the degree to which communities and biomes may become reassembled given the climate change velocities species will experience (Loarie et al., 2009). As dispersal propensity is often a dynamic trait influenced directly or indirectly by changing environmental conditions, this study reinforces the need to incorporate how changing abiotic conditions themselves will influence the ability of species to respond to future climate change (Clobert, 2001; Ronce, 2007; Walters et al., 2006).

## CONFLICTS OF INTEREST

The authors confirm that there are no known conflicts of interest and that the funders of this research had no input into this manuscript.

## AUTHOR CONTRIBUTION

**Andrew J. Prinster:** Conceptualization (equal); Data curation (lead); Formal analysis (supporting); Methodology (equal); Writing‐original draft (equal); Writing‐review & editing (equal). **Julian Resasco:** Conceptualization (equal); Data curation (supporting); Formal analysis (lead); Methodology (equal); Visualization (equal); Writing‐original draft (equal); Writing‐review & editing (equal). **César Roberto Nufio:** Conceptualization (equal); Data curation (supporting); Formal analysis (supporting); Funding acquisition (lead); Investigation (equal); Methodology (equal); Project administration (lead); Supervision (lead); Visualization (equal); Writing‐original draft (equal); Writing‐review & editing (equal).

## Supporting information

Table S1

## Data Availability

Data are available at: https://doi.org/10.5061/dryad.4qrfj6q8c
