# Supplementary material for: Weather variation affects the dispersal of grasshoppers beyond their elevational ranges
Source: Ecol Evol. 2020 Dec 2;10(24):14411–22. doi: 10.1002/ece3.7045 (PMC7771169; doi:10.1002/ece3.7045)
Supplement: Supplementary file 1 — Table S1 [file ECE3-10-14411-s001.docx]

**Table S1**. Fixed effects estimates from GLMMs with *Elevation* and *Year* as fixed effects. Year dropped from model in (b) due to model saturation.

a) Number of dispersers

|  | **Standardized**  **coefficient** | **SE** | **z-value** | **P-value** |
| --- | --- | --- | --- | --- |
| Temperature | 0.96 | 0.21 | 4.64 | 3.50 x 10^-6^ |
| Wind U-vector (2m) | 0.93 | 0.21 | 4.36 | 1.29 x 10^-5^ |
| Wind speed (80m) | -0.19 | 0.17 | -1.12 | 0.26 |

b) Species richness of dispersers

|  | **Standardized**  **coefficient** | **SE** | **z-value** | **P-value** |
| --- | --- | --- | --- | --- |
| Temperature | 0.36 | 0.11 | 3.19 | 0.001 |
| Wind U-vector (2m) | 0.39 | 0.13 | 3.11 | 0.002 |
| Wind speed (80m) | -0.02 | 0.10 | -0.23 | 0.821 |
